# Ancestry reported by white adults with cutaneous melanoma and control subjects in central Alabama

**DOI:** 10.1186/1471-2407-4-47

**Published:** 2004-08-13

**Authors:** Ronald T Acton, Ellen H Barton, William W Hollowell, Amy L Dreibelbis, Rodney CP Go, James C Barton

**Affiliations:** 1 Immunogenetics Program and Departments of Microbiology, University of Alabama at Birmingham, Birmingham, Alabama, USA; 2 Department of Medicine, University of Alabama at Birmingham, Birmingham, Alabama, USA; 3 Department of Epidemiology and International Health, University of Alabama at Birmingham, Birmingham, Alabama, USA; 4 Southern Iron Disorders Center, Birmingham, Alabama, USA

## Abstract

**Background:**

We sought to evaluate the hypothesis that the high incidence of cutaneous melanoma in white persons in central Alabama is associated with a predominance of Irish and Scots descent.

**Methods:**

Frequencies of country of ancestry reports were tabulated. The reports were also converted to scores that reflect proportional countries of ancestry in individuals. Using the scores, we computed aggregate country of ancestry indices as estimates of group ancestry composition. HLA-DRB1*04 allele frequencies and relationships to countries of ancestry were compared in probands and controls. Results were compared to those of European populations with HLA-DRB1*04 frequencies.

**Results:**

Ninety evaluable adult white cutaneous melanoma probands and 324 adult white controls reported countries of ancestry of their grandparents. The respective frequencies of Ireland, and Scotland and "British Isles" reported countries of ancestry were significantly greater in probands than in controls. The respective frequencies of Wales, France, Italy and Poland were significantly greater in controls. 16.7% of melanoma probands and 23.8% of controls reported "Native American" ancestry; the corresponding "Native American" country of ancestry index was not significantly different in probands and controls. The frequency of HLA-DRB1*04 was significantly greater in probands, but was not significantly associated with individual or aggregate countries of ancestry. The frequency of DRB1*04 observed in Alabama was compared to DRB1*04 frequencies reported from England, Wales, Ireland, Orkney Island, France, Germany, and Australia.

**Conclusion:**

White adults with cutaneous melanoma in central Alabama have a predominance of Irish, Scots, and "British Isles" ancestry and HLA-DRB1*04 that likely contributes to their high incidence of cutaneous melanoma.

## Background

The incidence of cutaneous melanoma in Alabama is high (16.9 and 8.6 per 100,000 men and women, respectively, in 1998) [1]. The average annual incidence rate in Alabama during 1996 was 15.5 per 100,000 for men and 8.8 per 100,000 for women, indicating that the incidence in men is increasing. This is consistent with reports that cutaneous melanoma is one of few cancers the incidence and mortality of which is increasing in white Americans [2]. Lifestyle, sun exposure, fair skin, light eye color, poor ability to tan, Northern European or Celtic ethnicity, family history of melanoma, benign nevi, various major histocompatibility complex alleles, and mutations in the p16 gene have been reported as risk factors for cutaneous melanoma [3-16]. HLA-DRB1*04 is significantly associated with cutaneous melanoma in white persons in Alabama [9] and Australia [11,12]. In addition, HLA-DRB1*04 was reported positively associated with cutaneous melanoma in white patients who had a high index of Celtic ancestry [14].

It has been hypothesized that the high incidence of melanoma in Alabama, a Sunbelt state with a mild climate, is due to the settlement of Alabama by a predominance of persons of Irish and Scots descent [8-10]. In the present study, we sought to evaluate this hypothesis by using questionnaires to obtain information about the countries of ancestry of the grandparents of white adults in central Alabama with cutaneous melanoma and of white control subjects from the central Alabama general population. Using data from the questionnaires, we evaluated the frequency of country of ancestry reports in cutaneous melanoma and control participants and computed country of ancestry indices to permit quantification and comparison of group ancestry data, as previously described [17]. We also compared HLA-DRB1*04 frequencies in participants with melanoma who reported "Celtic" and "non-Celtic" ancestry. The rationale for using country of ancestry information and HLA-DRB1*04 to identify persons who have increased risk to develop cutaneous melanoma is discussed.

## Methods

### General criteria for selection of study subjects

The performance of this work was approved by the Institutional Review Boards of the University of Alabama at Birmingham and Brookwood Medical Center. All subjects were adults (≥ 18 years of age) who were central Alabama residents; each identified himself/herself as white. Persons with melanoma were diagnosed in routine medical care in the interval 1981 – 2002, but were otherwise unselected. We excluded persons of sub-Saharan African or African American descent, because most of these persons have non-European ancestry and their incidence of cutaneous melanoma is much lower than in white persons in central Alabama [1,2].

### Melanoma probands

Cutaneous melanoma was diagnosed as previously described [9]. HLA-DRB1 typing was performed by either the microdroplet lymphocytotoxicity test using B-lymphocytes isolated by the nylon wool column procedure, or by low-resolution SSP using genomic DNA obtained from peripheral blood buffy coat, as previously described [9,18]. Further, we included only the first persons diagnosed to have cutaneous melanoma in respective families; they were designated as probands.

### Control subjects

Adult control subjects residing in central Alabama were recruited in two groups. The first group consisted of 83 spouses of persons with cutaneous melanoma and randomly recruited subjects. The second group consisted of 260 unselected volunteers who completed the present questionnaire (described below); they were recruited from hospital workers, employees of two universities, spouses of patients who attended a hematology/medical oncology outpatient clinic, and members of the general public encountered in two retail shopping malls. In both groups, we excluded persons who were known to be relatives of other study participants. We did not evaluate medical histories or perform physical examinations in control subjects. These data were pooled to yield a group of 343 unrelated controls, of whom 24 were eliminated because they did not know the country of ancestry of any of their grandparents (as indicated below). This left a group of 319 control subjects whose data were deemed evaluable for the present study. HLA typing was performed on some control subjects as indicated below.

### Questionnaire and interview design

A one-page questionnaire was designed to permit each study participant to indicate the countries of ancestry of his/her paternal and maternal grandparents. This method is identical to that previously validated in a study of the ancestry of hemochromatosis probands [17]; in part, this method was modified from previously reported methods by including only the country of birth of grandparents [6,14,19].

We defined aggregate country categories as the composite of reports from respective countries [17]. Reports based solely on association of family names with specific countries were tabulated as "Don't Know." Reports from participants for whom each of four grandparents were categorized as "Don't Know" were defined as inevaluable and were excluded from final analysis [17]. We did not evaluate relationships of country of ancestry to gender of the grandparents or to the paternal or maternal side of the participant's family [17].

### Frequencies of countries of ancestry reports

We tabulated the number of participants who reported specific countries of ancestry or aggregate country categories (as defined above) for one or more grandparents. This method is identical to that previously described [17]; in part, this method was modified from previously reported methods by including only the country of birth of grandparents [14,19].

### Country of ancestry indices

The questionnaire and interview reports from each participant were evaluated to yield individual country of ancestry scores that reflect proportional national ancestry, as previously described [17]. We also computed aggregate country of ancestry indices for cutaneous melanoma probands and control subjects as estimates of group ancestry composition. These indices were expressed as the quotient of total individual scores for respective countries and the total number of cutaneous melanoma probands or control subjects, as appropriate [17].

### Index of "Celtic" ancestry

An index of Celtic ancestry was computed in a manner similar to that used for country of ancestry indices. In the present analysis, persons who reported one or more grandparents whose country of origin was Ireland or Scotland were defined as "Celts." Other participants were defined as "non-Celts." We did not use grandparental surnames or maiden names to quantify the degree of Celtic ancestry as did previous investigators [14,19].

### Review of HLA-DRB1*04 phenotype frequencies in europe and australia

A manual and computerized literature search was performed to identify reports of HLA-DRB1*04 phenotype frequencies in white persons with or without melanoma in countries or areas of Europe and Australia that are known to have had Celt settlements The HLA-DRB1*04 phenotype frequency among white control subjects in central Alabama is approximately 0.2148, based on the combined data from two previous reports [9,20]. Thus, we tabulated only those reports from countries or regions in which the HLA-DRB1*04 phenotype frequency was > 0.2148. We excluded studies which reported HLA-DRB1*04 frequency estimates on control cohorts of fewer than 100 subjects.

### Statistical considerations

The dataset consisted of observations on 90 cutaneous melanoma probands and two groups of controls (83 interview subjects and 261 questionnaire responders, respectively). A computer spreadsheet (Excel 2000, Microsoft Corp., Redmond, WA) and a statistical program (GB-Stat, v. 8.0 2000, Dynamic Microsystems, Inc., Silver Spring, MD) were used to perform the present analyses. In a preliminary evaluation, we determined that the proportions of men and women, mean ages, country of ancestry reports, and frequencies of country of ancestry reports were not significantly different in the two control groups. Therefore, we pooled data from the two groups for comparison with those of melanoma probands. Frequencies of men and women, clinical abnormalities, and countries of ancestry were counted. General descriptive data are presented as percentages or mean ± 1 S.D. Comparisons between groups were tested for statistical differences using chi-square analysis, Fisher exact test or two-tailed Student *t *test, as appropriate. However, Student t-test can not be used to compare country of ancestry data groups in which all values are 0 (no variability). Accordingly, we arbitrarily assigned a country of ancestry datum of 0.25 for one person in each proband country group for which there were no actual country of ancestry reports. Student t-test was then performed using this modified data group; estimated p values from these tests are displayed in parentheses. A p value < 0.05 was defined as significant. Odds ratios (OR) were calculated as described by Woolf [21].

## Results

### Characteristics of cutaneous melanoma probands

There were reports from 90 evaluable probands (46 men, 44 women). Their mean age at the time of participation in the present study was 49 ± 16 years (range 19 – 80 years).

### Characteristics of control subjects

There were reports from 319 evaluable control subjects (128 men, 191 women). The percentages of men and women in the control group were similar to those in the cutaneous melanoma probands (p = 0.0621, chi square analysis). Their mean age at the time of participation in the present study was 43 ± 16 years (range 18 – 82 years). The mean age of the cutaneous melanoma probands was significantly greater than that of the control subjects (49 ± 16 years vs. 43 ± 16 years, respectively; p = 0.0016).

### General analysis of questionnaire and interview reports

No person declined to participate in the study, and there were no incomplete, equivocal, or unintelligible questionnaire or interview reports. Some participants reported that they were unaware of their ancestry due to adoption, family estrangement, or disinterest in genealogy. Ninety of 110 cutaneous melanoma probands (81.8%) and 319 of 347 control subjects (91.3%) provided reports for at least one of four grandparents; these differences were significant (p = 0.0026). Data from these participants were included for further analysis. Thus, there were reports from 90 evaluable cutaneous melanoma probands and 319 evaluable control subjects. Among evaluable cutaneous melanoma probands, the mean number of countries reported was 2.2 ± 1.1 (range 1 – 5 countries). The mean number of countries reported by evaluable control subjects was similar (2.4 ± 1.1 countries (range 1 – 7 countries); p = 0.2246).

### Frequencies of country of ancestry reports

These data are displayed in Table 1. Most participants reported European countries of ancestry. Frequencies of Ireland and Scotland country reports of ancestry obtained from cutaneous melanoma probands were significantly greater than those from control subjects (p = 0.0042, OR = 2.0 and p = 0.0015, OR = 2.2, respectively). The "Celtic" country reports of ancestry was also significantly higher in melanoma probands than in control subjects (Table 1). The frequency of "British Isles" and "Europe Not British Isles" ancestry reports tabulated in cutaneous melanoma probands were not significantly different than those in controls subjects. The respective frequencies of Wales, France, Italy, and Poland ancestry reported by cutaneous melanoma probands were significantly lower than those in control subjects (Table 1). 16.7% of country of ancestry reports from cutaneous melanoma probands and 23.8% in control subjects indicated "Native American" ancestry; these percentages were not significantly different (Table 1). The percentage of cutaneous melanoma probands who reported "Don't Know" for the countries of ancestry of one, two, or three grandparents was similar to that in control subjects (30.0% vs. 30.1%, respectively) (Table 1).

### Country of ancestry indices

These data are displayed in Table 2. The respective Ireland and Scotland indices in cutaneous melanoma probands were significantly greater than those in control subjects. The "Celtic" ancestry index was significantly higher in melanoma probands than in control subjects. The aggregate "British Isles" index was also significantly greater in melanoma probands (Table 2). The Wales, France, Spain, Austria, Italy, Poland, Russia, Sweden and "Europe Not British Isles" indices in probands were significantly lower than those in control subjects (Table 2). There were no other significant differences.

### HLA-DRB1*04 frequencies

HLA-DRB1 phenotypes were available for 63 of the 90 present cutaneous melanoma probands. We divided the probands for which we had both country of ancestry data and HLA-DRB1 phenotypes into "Celts" (n = 19) and "non-Celts" (n = 44), as defined above. There was no significant difference in the frequency of HLA-DRB1*04 in the two groups (0.5263 vs. 0.3636, respectively; p = 0.3551, OR = 1.9). Because this finding was unexpected and differed from that of previous reports [14] we then computed the frequencies of HLA-DRB1*04 in Alabama white cutaneous melanoma probands and in control subjects from two previous Alabama reports [9,20]. Thus, the frequency of HLA-DRB1*04 in 123 probands (38.2%) was significantly different from that in 340 controls (21.5%) (p = 0.0005, OR = 2.3).

### DRB1*04 phenotype frequencies > 0.2148 in Europe and Australia

We identified HLA-DRB1*04 phenotype frequency estimates of > 0.2148 from England, Ireland, Scotland, Wales, Orkney Islands, Brest, Germany, and Australia, areas populated predominantly by persons of Celtic ancestry [9,11,13,20,22]. These data and the results of studies in which HLA-DRB1*04 phenotype frequencies were assessed in melanoma patients are displayed in Table 3. The frequencies of HLA-DRB1*04 in these countries or regions are significantly higher than that in Alabama subjects (p ≤ 0.005) (Table 3).

## Discussion

The present results indicate that England, Ireland, Scotland and the aggregate "British Isles" are the countries of ancestry reported most frequently by cutaneous melanoma probands and control subjects in central Alabama. Germany is another country of ancestry often reported by the present study participants. These results are consistent with historical accounts of early migrations of persons of English, Irish and Scots descent into central Alabama [23-26], with the national associations of surnames recorded in Alabama Census returns for 1820 and 1830 [27], and with the present composition of the southern United States [25]. In U.S. Census 2000, country of ancestry information (maximum of two countries) was reported on a "long form" provided to one in six census participants, and was tabulated as numbers of country-specific reports [28]. Thus, the data of U.S. Census 2000 cannot be compared statistically with the results of the present study, but the percentages of European countries of ancestry of white Alabama residents compiled in both studies reveal similar trends. In the present study, the largest subgroups of reports in the "North, Central, and South American Countries" category are those of "U.S." or "American" ancestry. Some participants did not report or know the country of ancestry of their grandparents. These findings are also consistent with trends in the U.S. Census 1990 and U.S. Census 2000, in which the percentages of white Americans who report "American" ancestry are increasing, and the percentages of those who report various European countries of ancestry are decreasing [28,29].

"Native American" ancestry, especially Cherokee or Creek heritage, was reported by many of the present study participants, and this is consistent with accounts of early Alabama history [24,30-32] and with U.S. Census data on Alabama since 1820 [27-29]. However, the corresponding aggregate "Native American" frequency of country ancestry reports and country of ancestry indices were not significantly different in melanoma probands and control subjects. This supports the postulate that native American ancestry does not contribute significantly to the increased frequency of cutaneous melanoma in central Alabama.

The percentages of men and women were not significantly different in cutaneous melanoma probands and control subjects. Analyses of the two study groups indicate that age is not significantly correlated with country of ancestry indices. The mean age of cutaneous melanoma probands was significantly greater than that of control subjects. However, the mean number of countries reported by cutaneous melanoma probands and control subjects did not differ significantly. Thus it appears that diagnosis of cutaneous melanoma or greater age is not associated with greater interest or knowledge in personal ancestry, although this is unproven.

30.0% of cutaneous melanoma probands and 30.1% of control subjects did not know the country of ancestry of any of their four grandparents and were thus declared inevaluable. Many others did not know the ancestry of some of their grandparents. Some participants reported that they were unaware of their ancestry due to adoption, family estrangement, or disinterest in genealogy. Other participants could have been incorrect in their reporting. The percentages of evaluable cutaneous melanoma probands and control subjects who reported grandparents in the "Don't Know" category were similar. Altogether, it is unlikely that exclusion of subjects who did not know the country of ancestry of each of their grandparents would significantly change the outcomes of the present study. The overall trends in frequency of country reporting and country of ancestry indices in "British Isles" and "Europe Not British Isles" categories in the present study were similar. This suggests that uncertainty of participants about the exact degree of country of ancestry of some of their grandparents was probably not a significant contributor to the major conclusions of the present study. It is possible that control subjects who were included also had cutaneous melanoma. Nonetheless, it is unlikely that identification of a presumably small number of undiscovered control subjects with cutaneous melanoma would significantly change the conclusions of the present study.

Previous studies reported that Celtic ancestry is a risk factor for cutaneous melanoma, particularly when people of Celtic ancestry inhabit areas of high flux of ultraviolet radiation [4,6]. Persons of Celtic ancestry usually have fair skin, eyes and hair of light color, poor ability to tan, and tendency to burn easily after sun exposure [4,6]. Areas of the British Isles where persons have a high degree of Celtic ancestry include Wales, Cornwall, Scotland and Ireland [6]. We did not specifically ask about nor did we receive reports of Cornwall as a region of origin in the present study. Similarly, Wales was reported as a country of origin by none of the present probands and few of the control subjects. However, the frequency of country reports and ancestry indices for Ireland, Scotland, aggregate "British Isles," and combined Ireland and Scotland ("Celts") in the present melanoma probands was significantly greater than that in control subjects, consistent with the hypothesis that Celtic ancestry is a risk factor for cutaneous melanoma.

The present observations are consistent with those of another study in Alabama in which white persons with hemochromatosis, a disease thought to be of Celtic origin [33,34], reported a significantly greater Scotland and "British Isles" ancestry than controls [17]. The countries of ancestry reported was also consistent with the relative high frequency of C282Y, the major allele associated with hemochromatosis, in Alabama hemochromatosis probands compared to controls [17].

Using an index of Celtic ancestry that included grandparental surnames, maiden names, and country of birth to evaluate persons in Wales, other investigators observed that "high-scoring Celts" were significantly more likely to have Fitzpatrick skin type I or II (poor ability to tan, and tendency to burn easily after sun exposure) [35] than non-Celtic subjects [14,19]. Moreover, the frequency of HLA-DRB1*04 was significantly greater in "high-scoring Celts." These authors concluded that the increased risk of cutaneous melanoma and other types of skin cancer in persons of Celtic ancestry in Wales is due not only to paler skin, but also to HLA-DRB1*04 and associated immunologic factors.

Observations from previous studies demonstrate that the frequency of positivity for the HLA-DRB1*04 phenotype is significantly greater in Alabama cutaneous melanoma probands than in control subjects [8,9,20]. This is consistent with reports from Australia and Wales [11,12,14]. Further, previous reports from Alabama suggest that the subgroup of individuals who possess HLA-DRB1*04 are at increased risk for cutaneous melanoma, independent of eye color, hair color, amount of melanin in the skin, or ethnic origin [8]. However, the failure to reach statistical significance in the comparison of HLA-DRB1*04 phenotypes in the present 19 "Celts" and the 44 "non-Celts" is likely due to the small number of probands who reported Celtic ancestry and for whom HLA typing data were available.

Investigators in Texas and England reported that the HLA genotype DQB1*0301 influences either susceptibility to and/or severity of melanoma [15,36-38]. This conclusion is consistent with previous studies in which HLA-DRB1*04 was associated with melanoma, because HLA-DQB1*0301 is in linkage disequilibrium with HLA-DRB1*04 [37].

The highest frequencies of HLA-DRB1*04 in Europe occur in England, Wales, Ireland, the Orkney Islands, and the Brest area of France [22]. These geographic results could explain the significantly increased frequency of HLA-DRB1*04 in cutaneous melanoma probands from central Alabama, because the present melanoma probands had significantly higher Ireland, Scotland, and "British Isles" country of ancestry indices than Alabama control subjects.

Our observations and those of others suggest that certain HLA genotypes may be markers for Celtic ancestry [8,14]. It has been reported that HLA phenotypes other than HLA-DRB1*04 occur in association with melanoma in various white national or ethnic groups [13,15,36,39,40]. This could be explained in part by the variation of HLA phenotype frequencies in these white national or ethnic groups or by other genes within the major histocompatibility complex in linkage disequilibrium that also play a major role in mediation of immune responses. Thus, various HLA phenotypes could be markers for white national or ethnic groups that also possess certain physical characteristics and immune response genes that increase their risk for developing cutaneous melanoma.

## Conclusions

The present results support our hypothesis, and are also consistent with the association of cutaneous melanoma with Ireland, Scotland and "British Isles" ancestry, HLA-DR phenotypes, and estimations of a northern European somatic phenotype (eye color, hair color, amount of melanin in the skin) previously reported in white persons who reside in central Alabama [3,4,8,9]. The present observations also suggest that targeting white persons with relatively high "Celtic" or "British Isles" country of ancestry indices and HLA-DRB1*04 for cutaneous melanoma prevention and early diagnosis efforts would be an effective strategy to decrease the morbidity and mortality of this type of malignancy.

## Competing interests

None declared.

## Authors' contributions

RTA assessed the patients and participated in assessing the control population, conceived the study, participated in data collection, laboratory evaluation of the patients and controls, statistical evaluation, and wrote part of the manuscript. EHB participated in assessed the control population and in data collection. WWH participated in assessed the control population and in data collection. ALD participated in assessed the patient and control populations, in data collection and laboratory evaluation of the patients and controls. RCPG participated in conceiving the study. JCB participated in conceiving the study, assessing the control population, statistical evaluation and wrote part of the manuscript. All authors approved the final version of the manuscript.

## Pre-publication history

The pre-publication history for this paper can be accessed here:



## Figures and Tables

**Table 1:** Frequencies of Country of Ancestry Reports in Adult Whites in Central Alabama. *

Country	Melanoma Probands % (n)	Control Subjects % (n)	P Value^†^
England	46.7 (42)	43.9 (140)	0.6394
Ireland	62.2 (56)	45.1 (144)	0.0042
Scotland	44.4 (40)	27.0 (86)	0.0015
Ireland + Scotland ("Celts")	67.8 (61)	55.8 (178)	0.0417
Wales	0 (0)	4.7 (15)	0.0223
British Isles	73.3 (66)	75.6 (241)	0.6679
Native American	16.7 (15)	23.8 (76)	0.1493
Germany	24.4 (22)	33.5 (107)	0.1009
France	5.6 (5)	14.1 (45)	0.0287
Norway	1.1 (1)	1.6 (5)	0.6059
Netherlands	6.7 (6)	9.1 (29)	0.4678
Lithuania	0 (0)	0.3 (1)	0.7800
Czechoslovakia	1.1 (1)	0.9 (3)	0.6315
Spain	0 (0)	2.2 (7)	0.1730
Austria	1.1 (1)	2.2 (7)	0.4442
Belgium	1.1 (1)	0.3 (1)	0.3921
Denmark	1.1 (1)	1.6 (5)	0.6059
Finland	0 (0)	0.3 (1)	0.7800
Greece	2.2 (2)	1.3 (4)	0.3941
Hungary	1.1 (1)	0.6 (2)	0.5265
Italy	0 (0)	5.6 (18)	0.0102
Poland	0 (0)	5.0 (16)	0.0172
Romania	0 (0)	0.6 (2)	0.6079
Russia	0 (0)	2.5 (8)	0.1343
Sicily	0 (0)	1.6 (5)	0.2866
Sweden	1.1 (1)	4.4 (14)	0.1211
Switzerland	0 (0)	1.3 (4)	0.3685
Europe Not British Isles	63.3 (57)	62.7 (200)	0.9120
North, Central, and South American Countries	4.4 (4)	2.2 (7)	0.2057
Near and Middle East Countries	1.1 (1)	0.9 (3)	0.6315
African Countries	0 (0)	0.3 (1)	0.7800
Don't Know	30.0 (27)	30.1 (96)	0.9863

*These data include all reports by each evaluable study participant; there were 90 evaluable melanoma probands and 319 evaluable control subjects. The category "British Isles" was defined as the reports in England, Ireland, Scotland, and Wales categories. The category "Europe Not British Isles" is comprised of the composite data from the corresponding individual countries displayed in the present table. Participants who indicated specific "Native American" ancestry most frequently reported Cherokee and Creek descent. The "North, Central, and South America Countries" category (other than "Native Americans") included Canada, U.S., Virgin Islands, Colombia, and Brazil. The category "Near and Middle East Countries" included reports about grandparents from Iran, Lebanon, and Syria. The single observation in "African Countries" represents the Republic of South Africa. The "Don't Know" categories include data from participants who reported this category for one, two, or three grandparents. ^†^Chi-square or Fisher exact tests were used, as appropriate; p values < 0.05 were defined as significant.

**Table 2:** Country of Ancestry Indices in Adult Whites in Central Alabama. *

Country	Melanoma Probands	Control Subjects	p Value^†^
England	0.6559	0.7755	0.2896
Ireland	0.9573	0.6603	0.0150
Scotland	0.5924	0.3038	0.0031
Ireland + Scotland ("Celts")	1.5500	0.9640	0.0002
Wales	0	0.0674	(0.0005)
British Isles	2.2057	1.8069	0.0032
Native American	0.3184	0.2998	0.8493
Germany	0.4370	0.4610	0.8081
France	0.0889	0.1888	0.0001
Norway	0.0222	0.0230	0.9770
Netherlands	0.0903	0.1089	0.6909
Lithuania	0	0.0031	(0.9323)
Czechoslovakia	0.0037	0.0125	0.2967
Spain	0	0.0219	(0.0442)
Austria	0.0028	0.0214	0.0486
Belgium	0.0111	0.0031	0.4951
Denmark	0.0073	0.0167	0.3996
Finland	0	0.0063	(0.6110)
Greece	0.0203	0.0408	0.4491
Hungary	0.0222	0.0094	0.5863
Italy	0	0.1129	(0.0002)
Poland	0	0.0512	(0.0006)
Romania	0	0.0157	(0.3308)
Russia	0	0.0439	(0.0111)
Sicily	0	0.0392	(0.0667)
Sweden	0.0111	0.0674	0.0186
Switzerland	0	0.0157	(0.1400)
Europe Not British Isles	0.7169	1.2628	0.0001
North, Central, and South American Countries	0.1000	0.0533	0.4378
Near and Middle East Countries	0.0444	0.0408	0.9407
African Countries	0	0.0063	(0.6110)
Don't Know	0.5778	0.5235	0.6126

* These data include all reports by each evaluable study participant. The questionnaire and interview reports from each participant were evaluated to yield individual country of ancestry scores which reflect proportional national ancestry. For this, each participant was assigned 4.00 points divided equally among each of his/her 4 grandparents. If a participant indicated 2 or more countries of ancestry for a grandparent, the 1.00 point for that grandparent was equally divided among the respective countries to formulate the scores of individual participants. We also computed aggregate country of ancestry indices for melanoma probands and control subjects as estimates of group ancestry composition. These indices were expressed as the quotient of total individual scores for respective countries and the total number of melanoma probands or control subjects, as appropriate. The category "British Isles" was defined as the reports in England, Ireland, Scotland, and Wales categories. The category "Europe not British Isles" is comprised of the composite data from the corresponding individual countries displayed in the present table. Participants who indicated specific "Native American" ancestry most frequently reported Cherokee and Creek descent. The "North, Central, and South America Countries" category (other than "Native Americans") included Canada, United States, Virgin Islands, Colombia, and Brazil. The category "Near and Middle East Countries" included reports about grandparents from Iran, Lebanon, and Syria. The single observation in "African Countries" represents the Republic of South Africa. The "Don't Know" categories include data from participants who reported this category for one, two, or three grandparents. ^† ^Student *t *test cannot be used to compare country of ancestry data groups in which all values were 0 (no variability). Accordingly, we arbitrarily assigned a country of ancestry datum of 0.25 for one person in each proband country group for which there were no actual country of ancestry reports. Student *t *test was then performed using this modified data group; estimated values of p from these tests are displayed in parentheses.

**Table 3:** HLA-DRB1*04 Phenotype Frequencies in Central Alabama, European and Australian White Populations.

Country (City or Region)	HLA-DRB1*04 Frequency	
	
	Melanoma Patients (n)	Control Subjects (n)
USA (Central Alabama)	0.3820 (123)^‡^	0.2148 (340)^‡^
England (Manchester)	NA	0.3257 (786)^†^
Wales	NA	0.3651 (7,165)^†^
Ireland (Belfast)	NA	0.3422 (5,000)^†^
Ireland (Dublin)	NA	0.3063 (1,910)^†^
Orkney Island	NA	0.3556 (104)^†^
France (Brest)	NA	0.3933 (150)^†^
Germany (Munich)	0.2350 (98)^#^	0.2170 (847)^#^
Australia (Melbourne)	0.5590 (34)^¶^	0.3280 (210)^¶^

NA = Not available ^‡^Reported in references 9, 20 ^† ^Reported in reference 22. ^# ^Reported in reference 13 ^¶ ^Reported in reference 11
